# Author Correction: Condensate and superfluid fraction of homogeneous Bose gases in a self-consistent Popov approximation

**DOI:** 10.1038/s41598-024-70357-y

**Published:** 2024-08-22

**Authors:** Cesare Vianello, Luca Salasnich

**Affiliations:** 1 Department of Physics and Astronomy “Galileo Galilei”, University of Padova, Via Marzolo 8, 35131 Padua, Italy; 2 Padua QTech Center, University of Padova, Via Gradenigo 6/A, 35131 Padua, Italy; 3 Padova Section, National Institute of Nuclear Physics (INFN), Via Marzolo 8, 35131 Padua, Italy; 4 National Institute of Optics (INO) of National Research Council (CNR), Via Nello Carrara 2, 50127 Sesto Fiorentino, Italy

Correction to: *Scientific Reports* 10.1038/s41598-024-65897-2, published online 01 July 2024

The original version of this Article contained an error in Reference 9, which was incorrectly given as:

Leggett, A. J. Bose-Einstein-condensed gases with arbitrary strong interactions. *Rev. Mod. Phys.* 74, 063623 (2006).

The correct reference is listed below:

Yukalov, V. I. & Yukalova, E. P. Bose-Einstein-condensed gases with arbitrary strong interactions. *Phys. Rev. A*. **74**, 063623 (2006).

The original Article has been corrected.

